# Feasibility and acceptability of whole-body electromyostimulation in pulmonary rehabilitation for patients with chronic respiratory disease: a pilot study protocol

**DOI:** 10.1186/s40814-026-01883-x

**Published:** 2026-07-16

**Authors:** Atsuhito Nakazawa, Yuki Takahashi, Taichi Sano, Suzukaze Itakura, Yohei Arayama, Hikaru Machiguchi, Takamasa Morimoto, Takashi Fukushima, Takashi Niwa, Shigeru Komatsu, Takashi Ogura

**Affiliations:** 1 Department of Respiratory Medicine, Kanagawa Cardiovascular and Respiratory Center, 6-16-1, Tomioka-Higashi, Kanazawa-Ku Yokohama, Kanagawa 236-0051 Japan; 2 Department of Rehabilitation, Kanagawa Cardiovascular and Respiratory Center, 6-16-1, Tomioka-higashi, Kanazawa-ku Yokohama, Kanagawa 236-0051 Japan

**Keywords:** Whole-body electromyostimulation, Pulmonary rehabilitation, Chronic respiratory disease, Feasibility study, Pilot study, Exercise training, Skeletal muscle, Acceptability

## Abstract

**Background:**

Evidence on the effectiveness of electromyostimulation (EMS) has been reported in pulmonary rehabilitation, but the benefits have been limited in chronic obstructive pulmonary disease, and clinical trials in advanced idiopathic pulmonary fibrosis were not considered feasible. Recently, whole-body EMS (WB-EMS) has been developed and applied in the area of non-medical commercial application. To date, there are no reports on the application of medical WB-EMS for respiratory diseases. The aim of this study is to investigate the feasibility and acceptability of WB-EMS as an adjunct to pulmonary rehabilitation in patients with chronic respiratory disease.

**Methods:**

This is a protocol for a pilot, single group, feasibility trial. The e-skin is an EMS suit that can simultaneously load the muscles of the entire body (Xenoma Inc., Tokyo, Japan). Participants will conduct one session per day, once a week for 4 weeks. They perform resistance exercise trainings during EMS under the supervision and guidance of a trained physiotherapist or respiratory physician as a trainer. A physiotherapist controls the HUB attached to the suit with a dedicated mobile application. Each setting of EMS is determined by each segment of electrical stimulation and meets the recommended regimen and guideline criteria for WB-EMS operation. Outcome measures will include Modified Medical Research Council Dyspnoea Scale, pulmonary function test, health-related quality of life, exercise tests including six-minute walk test and one-minute sit-to-stand-test, Barthel Index, muscle strength, body composition analysis, and blood tests such as muscle markers and renal function markers. To assess the feasibility and acceptability of the intervention, participant adherence, retention, and perceived burden will be evaluated.

**Discussion:**

WB-EMS can stimulate both type I and type II muscle fibers and may help counteract preferential type II fiber atrophy frequently observed in CRD. This pilot study focuses on verifying the feasibility and acceptability of WB-EMS as an adjunct to pulmonary rehabilitation. By providing a physiologically distinct, time-efficient training modality that minimizes cardiopulmonary load, WB-EMS could become a promising rehabilitation option for patients with limited exercise tolerance.

**Trial registration:**

UMIN-CTR, UMIN000057270. Retrospectively registered 12 March 2025.

**Supplementary Information:**

The online version contains supplementary material available at https://doi.org/10.1186/s40814-026-01883-x.

## Background

Patients with chronic respiratory disease (CRD) present with subjective symptoms such as dyspnea and cough, and as the disease progresses, they suffer from complications, significant loss of weight, and reduced activities of daily living [1]. The effectiveness of pulmonary rehabilitation has been reported for patients with CRD and is expected to improve subjective symptoms such as dyspnea, exercise tolerance, and health-related quality of life (HRQoL) [2, 3]. Pulmonary rehabilitation (PR) is a comprehensive multidisciplinary intervention, and exercise therapy in particular is one of its major components [4]. However, the full benefit of exercise therapy cannot be achieved, for example, when exercise therapy cannot be implemented for various reasons, and when the disease progresses and becomes more severe.

Electromyostimulation (EMS) applies an electric current to the muscles to cause them to contract, providing load without the use of heavy equipment, which may reduce the risk associated with conventional resistance training [5]. And it allows for applying relatively high muscle loading with less stress on cardiopulmonary function, bones, and tendons [6–9]. EMS may provide additional benefits when combined with active exercise compared to exercise alone, particularly in individuals with limited exercise tolerance. However, the effectiveness of EMS with passive exercise—defined as externally assisted joint movement without voluntary muscle contraction—is unclear [5], and evidence regarding EMS combined with active resistance exercise in patients with CRD remains limited. It has been utilized in the nursing care and rehabilitation fields and has proven effective for patients with muscle weakness and reduced activities of daily living [10, 11].

In PR, EMS has been used, and evidence on its effectiveness has been reported, although the number of studies remains limited. A meta-analysis demonstrated a significant improvement in quadriceps strength, muscle mass, and exercise capacity [10]. Improved exercise capacity and decreased dyspnea on exertion have been reported in patients with chronic obstructive pulmonary disease (COPD) [12]. Home-based PR incorporating neuromuscular electrical stimulation has been reported to improve exercise capacity in patients with severe COPD [13, 14]. In a randomized placebo-controlled feasibility study in patients with advanced idiopathic pulmonary fibrosis, because of recruitment difficulties, a definitive clinical trial using the protocol in this population was not considered feasible [15]. However, these EMS devices only provide electrical stimulation to a certain part of the body, such as the lower limbs, and the effect may be limited.

Whole-body EMS (WB-EMS) has been developed and applied in recent years as an option that can load muscles throughout the body. WB-EMS can simultaneously stimulate large muscles in the upper and lower extremities and buttocks as well as the chest, abdomen, and back [16]. Guidelines for the safe and effective use of WB-EMS also exist [17]. New evidence on WB-EMS is emerging, but there are currently no reports yet on patients with CRD. Training muscle groups throughout the body with electrical stimulation is expected to improve not only exercise tolerance but also respiratory muscles, an effect not seen in conventional PR.

The objective of this study is to investigate the feasibility and acceptability of WB-EMS as an adjunct to PR in patients with CRD.

## Methods

### Trial design and setting

This is a protocol for a pilot, single-arm, feasibility study. This study was designed as a pilot study to assess the feasibility of implementing WB-EMS in patients with CRD. Pilot studies are commonly conducted to evaluate the feasibility of study procedures before larger confirmatory trials [18]. Because the primary aim of this study is to evaluate the feasibility and acceptability of implementing WB-EMS in patients with CRD, a single-arm design was considered appropriate. This exploratory approach allows assessment of recruitment, adherence, and safety monitoring prior to the development of a future controlled trial. The study has been approved by the ethics committees at Kanagawa Cardiovascular Respiratory Center (KCRC-23-0010), in Yokohama, Japan. This is an investigator-initiated study without external funding or commercial sponsorship and is conducted under the responsibility of the investigators at this institution.

### Participant eligibility

Potential participants will be identified among outpatients referred to the PR clinic. Patients will be deemed eligible for the study if they are at least 18 years old, have a confirmed diagnosis of CRD, clinically stable, and have no supervised PR within the past 6 months (to avoid carry-over effects); unsupervised home exercise is not an exclusion. After eligibility screening and informed consent, those who opt in will enter the WB-EMS feasibility program; those who opt out will receive standard PR without delay. People with CRD will be excluded if they (i) have a marked exercise-induced desaturation; (ii) have a difficulty performing exercise due to severe musculoskeletal disorders or neurological disabilities that preclude safe training; (iii) have cardiac disease that is at risk with exercise; (iv) violates precautions for EMS suit use (neuromuscular problems, skin problems such as hypersensitivity or sensory disturbance, using a pacemaker or other medical electrical device, having a tumour, etc.); (v) have visual or hearing problems; (vi) have previously been advised by their physician to avoid exercise due to serious medical conditions; (vii) are unable to understand the exercise sufficiently; (viii) have a diagnosis of cognitive dysfunction.

### Recruitment and consent

Potential participants will be identified among outpatients referred to PR at the Kanagawa Cardiovascular Respiratory Center. If they meet the inclusion criteria, a PR physician will provide a full explanation of the study procedures, risks, and benefits. Written informed consent will then be obtained from each participant prior to enrollment. The study will be conducted in accordance with the ethical principles outlined in the Declaration of Helsinki. After consent, participants will complete baseline assessments and receive nutritional guidance.

### WB-EMS intervention

#### EMS suit

The commercially available WB-EMS suit “e-skin” (Xenoma Inc., Tokyo, Japan) can simultaneously load the muscles of the entire body, and is used not only for daily use but also recently as a tool for clinical research on WB-EMS [19, 20]. The suit is made of stretchy material and comes in six different sizes to fit the body and allow for flexible training. Since the electrodes must adhere to the inner shirt and inner pants, it is important to select the appropriate size of the suit, and three sizes exist for them. Physiotherapist controls the HUB attached to the suit with a dedicated mobile application (app). The following are the independent control areas of the e-skin: pectoralis major, right and left arm (biceps and triceps), abdominals and obliques, back (latissimus dorsi, trapezius and erector spinae), buttocks (gluteus maximus), right and left leg (quadriceps and biceps femoris).

#### EMS suit training program

All training sessions will be conducted under WB-EMS suit stimulation. No conventional resistance exercise was performed in addition to EMS. The basic flow from the reception desk is like that of regular PR, but this program requires a change of clothes into the EMS suit. Participants must remove all clothing, including underwear, and wear the EMS suit over an inner shirt and pants. Participants will conduct one session per day, once a week for 4 weeks. This weekly frequency is consistent with recommendations for the initial 8–10 weeks of EMS training, which are considered necessary for adequate recovery and adaptation [17]. The intervention period was set at 4 weeks because the primary objective of this pilot study was to evaluate feasibility and acceptability rather than the effects of a longer-duration training program. A shorter intervention period was also considered appropriate to minimize participant burden and maintain flexibility for subsequent standard PR within routine clinical practice.

**Fig. 1:**
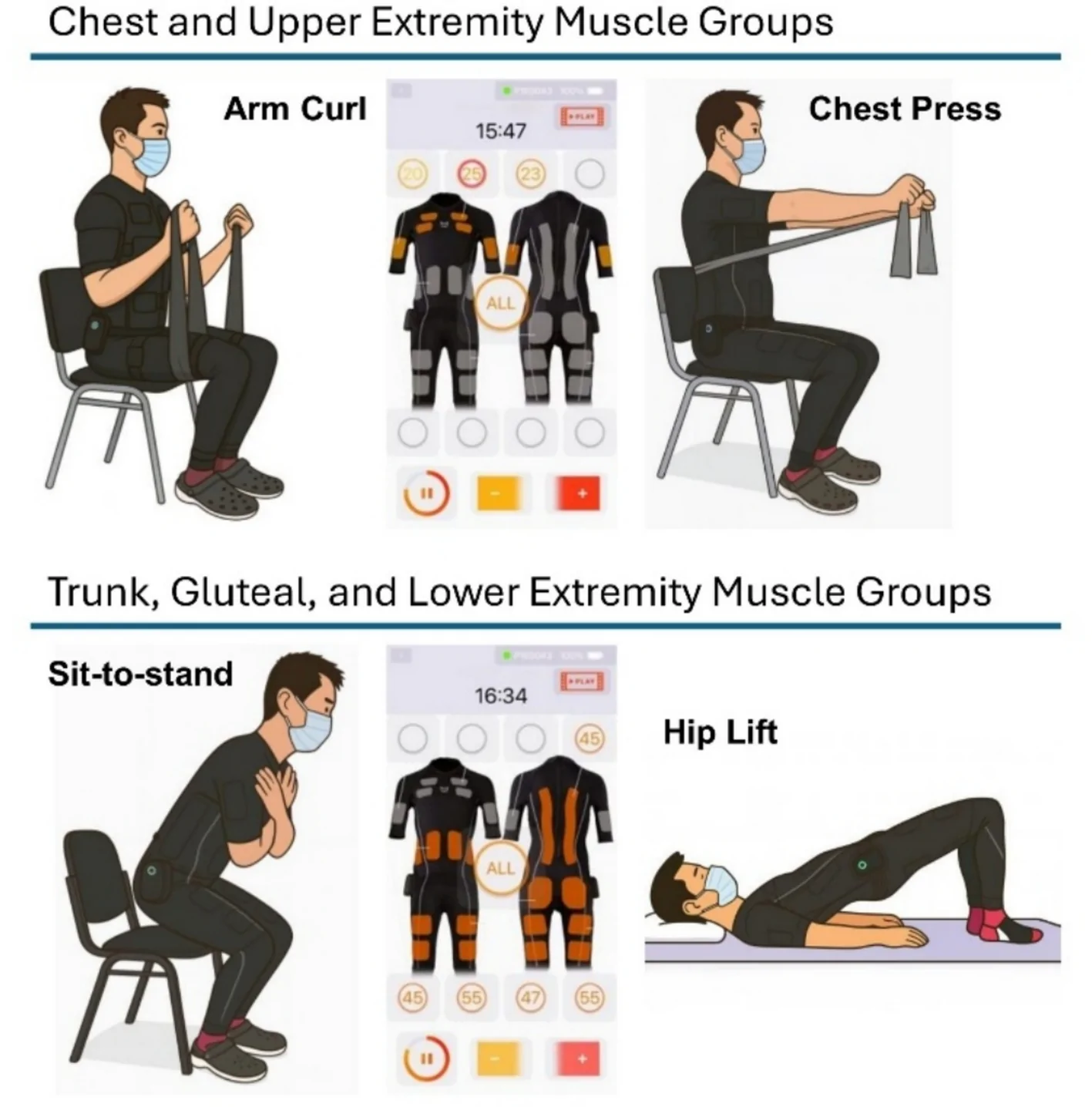
Resistance exercise training with WB-EMS suit and screenshots of the application in action. Elastic bands were used to provide adjustable resistance during the exercises. The Hub device is positioned on the outer side of the right hip and emits a flashing green light during operation. The accompanying screenshots illustrate the mobile application interface used to control the system. On the display, electrodes are highlighted in orange, indicating that electrical stimulation is in progress and that the stimulation can be adjusted. WB-EMS: whole-body electromyostimulation

They perform resistance exercise training under the supervision and guidance of a trained physiotherapist or respiratory physician as a trainer. The trainer should always remain in close proximity to the participant, ensuring a mandatory 1:1 trainer–participant ratio for safety supervision. They will perform a total of 4 exercises: arm curl, chest press, sit-to-stand, and hip lift while wearing an EMS suit. The program consists of two sets of upper body exercises including arm curl and chest press, and two sets of core and lower body exercises including sit-to-stand and hip lift. (Fig. 1) The intensity of the electric current during exercise is set to a level where the subject feels the muscular contraction caused by the electric current stimulation and does not feel pain. The load is set to the equivalent of 4–5 on the modified Borg scale (0–10) for perceived exertion related to EMS stimulation, using their own body weight or an elastic band. If the suit is loose and does not fit tightly, use an elastic band to stabilize the electrode's contact with the skin. The stimulation time per session is set to 20 min, following the operational guidelines for WB-EMS use in healthy populations [17]. While these recommendations are not based on clinical populations, we adopt them as a standardised setting, and the present study aims to evaluate the feasibility and acceptability of this protocol in patients with CRD. During exercise, participants should be monitored to maintain oxygen saturation (SpO_2_) of at least 90%. For patients already prescribed long-term oxygen therapy, supplemental oxygen will be used as usual. For patients not on oxygen therapy, if desaturation occurs, exercise will be paused and supplemental oxygen will be provided only if clinically indicated. Participants will perform one supervised WB-EMS session per week for four consecutive weeks (total of four sessions). After completion of the initial 4-week intervention period, participants who wish to continue and are able to tolerate the intervention may undergo up to two additional weekly sessions, for a maximum total of six sessions.

This feasibility study does not replace standard PR. Participation is voluntary, and declining or withdrawing will not affect access to PR. If WB-EMS is not tolerated, proves infeasible, or if the patient requests withdrawal, participation in the study will be discontinued and the patient will proceed to standard PR according to routine clinical care. After study completion, participants may also undergo standard PR as appropriate. Usual pharmacological treatment and routine clinical care for CRD will be continued throughout the study. Concurrent participation in standard PR during the WB-EMS intervention period will not be permitted.

#### EMS suit settings

The EMS parameters were not arbitrarily chosen by the investigators. Instead, they follow the pre-programmed “low-intensity” protocol provided by the manufacturer of the WB-EMS suit, which is widely applied in fitness and training practice. A low-intensity WB-EMS program was selected to improve tolerability and feasibility during this pilot intervention. This protocol uses a stimulation frequency of 85 Hz, a pulse width at 180 microseconds, and the stimulation cycle is set to 1 s on/1 s off. Stimulation intensity is adjusted individually at the initial evaluation (app score range: 0–100; score 1 equivalent to 2 mA). These settings follow the recommended regimens and guideline criteria for WB-EMS operation [17, 21, 22]. For safety reasons, we selected the low-intensity program, which is recommended for EMS novices and clinical populations. Each setting is determined for each segment of electrical stimulation during the first session. The initial set values are then used as reference values from session 2 onwards, with adjustments allowed according to individual tolerance. Slight variations in current tolerance are expected even under the same nominal settings. To prevent EMS-induced rhabdomyolysis due to excessively high current intensity, the maximum individual tolerance should be carefully evaluated and determined by the perceived exertion related to EMS stimulation, assessed with the modified Borg scale (0–10), which has been widely adopted in previous reports [23–26].

The intervention is conducted using the official WB-EMS device and its dedicated app provided by the manufacturer. Prior to the start of the program, the PR staff receive on-site training from an official trainer dispatched by the manufacturer. Through this training, staff are instructed in correct device operation and safety precautions, and the procedures are formally approved by the manufacturer. Although this is not a certificate-based qualification course, the staff complete the official training process and are authorized by the manufacturer to deliver the WB-EMS sessions in this study.

#### EMS suit management

The inner shirt and pants and EMS suit used should be taken home by the participant, laundered at home, and brought to the next training session. Washing and dehydration in an automatic washing machine is allowed, but use of a dryer should be avoided. Participants will not be held liable for any malfunctions of these dressing items or related equipment, but they are asked to handle them with care. As a rule, the EMS suit is collected after the program. Explanations within the scope of general operation regarding the EMS suit will be added orally if necessary.

### Outcome measures

Participants’ demographic information will be collected. Outcome measures will be assessed at two time points: baseline (prior to the first WB-EMS session) and after completion of the 4-week training program. At baseline, assessments will include modified MRC, pulmonary function test, health-related quality of life (St. George’s Respiratory Questionnaire, MOS 36-Item Short-Form Health Survey, Visual Analog Scale-8, Hospital Anxiety and Depression Scale), exercise tests, Barthel Index, muscle strength, and body composition analysis. After 4 weeks, re-evaluations will be performed for exercise tests, Barthel Index, muscle strength, and body composition analysis. Measures related to the study aim will be summarized based on exercise logs and interviews with participants as well as the results of pre- and post-training program assessments. Measures related to the efficacy of EMS suit training program will be compared before and after measures that will be made of body composition, muscle strength, exercise capacity, and exercise-induced desaturation. Outcome measurements will be conducted by trained clinical staff according to standardised procedures used in routine PR. Data will be recorded in dedicated case report forms and checked by the investigators to ensure completeness and accuracy.

### Primary outcomes

#### Feasibility and acceptability

To assess feasibility of the study protocol, dropouts (including reasons for withdrawal) and adherence will be investigated. The adherence rate was assessed as the ratio of the number of sessions undertaken, compared to the total number of those scheduled. A dropout rate of < 20% and adherence to ≥ 75% of scheduled sessions will be considered acceptable. Acceptability of the intervention will be explored through participant feedback during the training sessions, including perceived comfort of the WB-EMS suit, tolerability of electrical stimulation intensity, willingness to continue the program, and reasons for discontinuation if applicable. These data will be descriptively summarized to provide insight into the acceptability of WB-EMS in this population.

### Secondary outcomes

#### Body composition

Body composition analysis is measured by bioelectrical impedance analysis using InBody720 (Inbody Japan Inc., Tokyo, Japan). This can estimate body fat and muscle mass noninvasively and in a short time by calculating the resistance of low-level electrical currents flowing throughout the entire body. This method can also evaluate the body in five body segments (arms, legs, trunk).

#### Muscle strength

Muscle strength will be quantitatively assessed using the Micro Total Analysis System F-1 (Anima Inc., Tokyo, Japan). Muscle groups commonly evaluated in Manual Muscle Testing will be assessed bilaterally, including the biceps brachii, triceps brachii, knee extensors, hamstrings, and gluteus maximus muscles. Muscle strength values will be recorded in kilogram-force according to the manufacturer’s standard procedures.

#### Exercise capacity

Six-minute walk test (6MWT) and one-minute sit-to-stand-test (1MSTST) will be performed as an exercise test. The 6MWT is a submaximal exercise test used to measure the exercise capacity. It also evaluates the effectiveness of therapeutic intervention and allows for a non-specific, integrated assessment of the muscles and other systems during walking. This test is performed in compliance with the statements of the International Society using a straight-line distance of 30 m [27]. The 1MSTST is characterised as the exercise test using a standard chair (height 40 cm) with a flat seat and no armrests. Variables such as the walked distance on 6MWT and the number of repetitions on 1MSTST will be measured during the tests.

#### Exercise-induced desaturation

SpO_2_ will be continuously monitored during both exercise tests, namely the 6MWT and 1MSTST. Changes between baseline and nadir of SpO_2_ will be measured for the assessment of exercise-induced desaturation.

#### Safety

All adverse events related to this study will be collected and documented. Safety monitoring includes routine symptom checks performed in outpatient PR, where patients are asked about excessive pain, discomfort, or changes in urine color. These are assessed through patient self-reports and investigator interviews at each session. In addition, subjective exertion related to EMS stimulation is assessed using the modified Borg scale (0–10).

Blood tests assessing muscle-related markers and renal function will be performed at ≥ 72 h after the first WB-EMS session, corresponding to the expected peak of creatine kinase levels, to evaluate the risk of rhabdomyolysis and acute kidney injury [17]. In principle, blood sampling will be conducted at the time of Visit 2 (session 2) or thereafter. However, to minimize patient burden and integrate with routine clinical care, blood sampling may be performed earlier or later within this time window, provided that at least 72 h have elapsed after the initial session. In such cases, study-related measurements may be incorporated into clinically indicated blood tests at the discretion of the treating physician.

If abnormal laboratory findings or clinical symptoms suggestive of adverse events occur, participants will undergo additional clinical evaluation and follow-up blood testing as appropriate. Decisions regarding continuation, temporary suspension, or discontinuation of WB-EMS will be made comprehensively based on laboratory findings, symptoms, and overall clinical condition at the discretion of the treating physician. In the event of a serious adverse event, the intervention will be discontinued immediately, and appropriate medical evaluation and treatment will be provided according to standard clinical practice.

Events will be categorized according to standardized definitions of adverse events and serious adverse events.

### Figure: study schedule

The overall study schedule, including enrollment, intervention, and assessment time points, is illustrated in Table 1 in accordance with the SPIRIT recommendations.

**Table 1:** Participant timeline: schedule of enrollment, interventions, and assessments

	Trial period						
	Enrollment		Intervention (Weeks 1–4)				Post-intervention
Timepoint	*-t*_*i*_ to 0	0	*t*_*1*_	*t*_*2*_	*t*_*3*_	*t*_*4*_	*t*_*x*_
Enrollment:							
Eligibility assessment	X						
Informed consent	X						
Intervention:							
WB-EMS sessions (1 session/week)			X	X	X	X	
Assessments:							
Baseline assessments		X					
Safety monitoring			X	X	X	X	
Blood sampling (≥ 72 h after session 1; typically at or after t_2_)				X			
Outcome assessments							X

* − t1* screening, *t0* baseline, *t1–t4* intervention visits, *tx* post-intervention assessment

Blood sampling may be performed earlier or later within the predefined window (≥ 72 h after session 1), depending on clinical circumstances

### Data management and analyses

All collected data will be recorded in case report forms and entered into a secure electronic database by the investigators. The entered data will be checked for completeness and consistency by the research team. Personal identifiers will be removed prior to analysis, and the dataset will be stored securely at the study institution with access restricted to the study investigators.

Given the small sample size and the non-pharmacological nature of the intervention, a formal data monitoring committee has not been established. Study conduct and safety monitoring will be overseen by the investigators. No interim analyses are planned due to the exploratory nature of this pilot study. Decisions regarding continuation or termination of the study will be made by the investigators based on participant tolerability, adverse events, safety, and overall clinical condition. Formal trial monitoring will not be conducted due to the small scale and single-center nature of this pilot study. Study conduct will be supervised by the investigators, and adherence to the study protocol will be reviewed throughout the intervention period.

The analysis will be performed on participants who have completed the baseline assessments and have had at least one session. The data of discontinued cases will be evaluated using the values actually measured up to the point of discontinuation, regardless of the time of discontinuation. Missing data will not be imputed due to the exploratory nature of this pilot study, and analyses will be conducted using available data. The measurements were presented as mean and standard deviation for continuous variables, as median and interquartile ranges for non-normally distributed data, and as counts and percentages for categorical variables.

As this is a pilot single-arm feasibility study, the primary analyses will focus on descriptive evaluation of feasibility outcomes, including recruitment rate, adherence, retention, tolerability, and adverse events. Continuous variables will be summarized using descriptive statistics. Exploratory pre-post changes in clinical outcomes may be assessed using the Wilcoxon signed-rank test, and exploratory associations between baseline clinical characteristics and outcome measures may be evaluated using Spearman’s rank correlation coefficient. These analyses will be considered exploratory and hypothesis-generating in nature, given the small sample size, heterogeneous study population, and non-comparative design. Statistical software will be used (SPSS Inc, Chicago, IL, USA). *P*-value < 0.05 was considered as statistically significant.

### Sample size calculations

During the 2 years prior to study initiation, the annual number of new referrals for outpatient pulmonary rehabilitation at our institution was 39 in 2021 and 34 in 2022. Among these, 13 and 9 patients, respectively, met the eligibility criteria for this study. Based on these figures, we estimate that approximately 30–40 new patients with chronic respiratory disease will continue to be referred annually, with about 30% expected to meet inclusion criteria and provide written informed consent. Considering the program duration of approximately 6 weeks per participant, the target sample size of 12 patients was deemed realistic and appropriate for a pilot feasibility study. This sample size is consistent with methodological recommendations suggesting that approximately 12 participants per group are adequate for pilot studies aimed at assessing feasibility and informing the design of future trials [28, 29].

### Patient and public involvement

Patients and the public were not involved in the design, conduct, reporting, or dissemination plans of this study. The results of this study will be disseminated through publication in peer-reviewed journals and presentations at scientific conferences. The study results will also be reported in the University hospital Medical Information Network Clinical Trials Registry in accordance with registry requirements.

### Trial progress

Recruitment is ongoing. At the time of manuscript submission, seven participants have successfully completed the 4-week WB-EMS program, which includes pre- and post-intervention assessments. The target sample size is twelve participants as stated in the protocol. Since each participant requires a minimum of 6 weeks from baseline evaluation to post-intervention assessment, the remaining five participants are expected to complete the program within approximately eight additional months. Accordingly, the last patient/last visit is anticipated by November 2026.

Submission of this protocol was delayed due to a prolonged review process (approximately 10 months) at a previous submission, which resulted in a delay in public disclosure of the protocol. Nevertheless, the trial remains in progress and far from completion, and publication of the protocol at this stage is expected to enhance transparency, reproducibility, and constructive feedback from the scientific community. Participant retention will be promoted through supervised sessions and clinical monitoring. Data collected prior to withdrawal will be retained for analysis whenever possible.

### Trial registration

The study protocol was retrospectively registered with the University Hospital Medical Information Network Clinical Trials Registry (UMIN-CTR: UMIN000057270). Protocol version 1.0, dated November 2023.

## Discussion

To date, there have been no published reports on the application of medical WB-EMS for respiratory diseases. The recently updated guideline on WB-EMS is not a mandatory standard or a dedicated clinical practice guideline; rather, it only provides recommendations regarding safety and effectiveness in the context of non-medical commercial WB-EMS use [17]. In contrast, the National Institute for Health and Care Excellence guideline for COPD recommends neuromuscular electrical stimulation of the quadriceps as an adjunct to physical exercise, reflecting the clinical importance of lower limb muscle weakness in this population, but no recommendations have yet been made for WB-EMS [30]. Thus, verification of the feasibility and acceptability of WB-EMS in patients with CRD represents an important and novel area of investigation for future PR practice.

Despite the well-established benefits of PR, many patients with CRD remain unable to perform sufficiently intensive exercise because of severe dyspnea, ventilatory limitation, and early fatigue. These limitations may reduce the effectiveness of conventional exercise training and highlight the need for alternative or adjunctive strategies that can stimulate skeletal muscles with a lower cardiopulmonary burden while supporting participation in rehabilitation exercise. In this context, WB-EMS may represent a promising approach, as it allows simultaneous activation of multiple muscle groups while requiring relatively low voluntary effort.

From a physiological perspective, WB-EMS directly depolarizes motor neurons, resulting in the recruitment of both type I and type II muscle fibers [23, 25, 31]. EMS can activate both type I and type II muscle fibers; however, in patients with CRD, high-threshold motor units innervating type II fibers are preferentially recruited [32, 33]. Type II fibers are generally more susceptible to atrophy and functional impairment than type I fibers, suggesting that WB-EMS may help compensate for these fibre-type–specific deficits. Repeated EMS sessions are expected to enhance protein synthesis, improve peripheral muscle strength, and increase endurance capacity by promoting microcirculation and oxidative metabolism [23, 25, 31]. Reliable stimulation of the upper body and trunk muscles may also provide indirect benefits for respiratory muscles by improving chest wall mechanics and postural stability [34].

One of the main attractions of WB-EMS training is its ability to provide high training intensity within short sessions, thereby reducing the overall time burden for patients. Compared with conventional high-intensity resistance exercise in nonathletic healthy subjects, WB-EMS training has demonstrated comparable improvements in body composition and muscle strength, suggesting that it can be considered a time-efficient alternative [35, 36]. This time efficiency is particularly relevant for patients with CRD, who often experience severe shortness of breath and find long-duration exercise programs difficult and burdensome. By reducing the length and intensity of voluntary exertion, WB-EMS may help alleviate training-related discomfort, enhance motivation, improve adherence, and ultimately augment the overall benefits of PR.

Another attractive feature of WB-EMS is that stimulation intensity and targeted muscle groups can be adjusted using a dedicated mobile application. This flexibility allows individualized adjustment of stimulation according to patient tolerance and clinical condition, which may be particularly advantageous in patients with CRD who often present with heterogeneous symptoms and physical limitations. In addition, gradual adjustment of stimulation intensity may facilitate safe implementation in PR settings.

On the other hand, systemic factors in CRD, such as chronic inflammation, malnutrition, and ventilatory limitation, may attenuate these desired effects [37]. In addition, patients with CRD, who often experience weight loss and body habitus changes, face practical challenges when selecting a suit that fits their physique. If a participant is only thin, electrode contact can be reinforced with elastic bands; however, if the stimulation site is positioned inappropriately, the intended effect may not be achieved. Therefore, the process of choosing a WB-EMS suit that most closely resembles the participant’s body shape may represent a potential limitation of this study. In addition, although WB-EMS is generally considered to impose less cardiopulmonary burden than conventional exercise, transient discomfort or a temporary increase in perceived breathlessness cannot be completely excluded, particularly when trunk or respiratory-related muscles are stimulated. For this reason, all participants are carefully informed of both the potential risks and expected benefits prior to enrolment, and sessions are closely monitored to ensure safety. These disease-related limitations and the potential for transient discomfort highlight the importance of careful safety management. Therefore, the present study incorporates multiple precautionary strategies to ensure the safe implementation of WB-EMS in clinical practice.

Safe application of WB-EMS in patients with CRD requires careful patient selection, monitoring of comorbidities, and close observation of physical condition. Although EMS allows for a variety of stimulus modulations, it should always be applied according to the clinical situation. The perception of electrical stimulation varies among individuals, and as the session progresses, stimulus intensity needs to be frequently adjusted to account for muscle adaptation and the pronounced repeated bout effect [38–41]. With reference to a mini-review on potential side effects [16], we have designed this protocol to emphasize “safety first.” In this study, serum markers are assessed in all participants to detect exercise-induced muscle damage and inflammatory responses [42, 43]. The 4-week intervention period is intended as a familiarization and conditioning phase, minimising the risk of adverse events [17]. In addition, when a participant presents with coexisting cardiovascular disease, the cardiologist or attending physician is consulted, even if the condition does not meet the exclusion criteria. Taken together, these precautions ensure that all foreseeable risks are considered when applying WB-EMS in a medical setting.

In summary, WB-EMS represents a promising adjunct to PR for patients with CRD. By providing a physiologically distinct form of muscle activation, it may achieve benefits not obtained in conventional programs. This pilot study will evaluate the feasibility and acceptability of WB-EMS, aiming to demonstrate its potential as a new rehabilitation option that could broaden indications across diverse clinical backgrounds. Establishing these fundamental aspects is an essential step towards considering WB-EMS as a future component of comprehensive PR.

## Supplementary Information

Supplementary Material 1.

## Data Availability

The datasets generated and analysed during the current study will be available from the corresponding author on reasonable request.

## Abbreviations

1MSTST: One-minute sit-to-stand-test
6MWT: Six-minute walk test
COPD: Chronic obstructive pulmonary disease
CRD: Chronic respiratory disease
HRQoL: Health-related quality of life
PR: Pulmonary rehabilitation
SpO_2_: Oxygen saturation
WB-EMS: Whole-body electromyostimulation
